# Nitrosative stress triggers microtubule reorganization in *Arabidopsis thaliana*

**DOI:** 10.1093/jxb/eru194

**Published:** 2014-05-06

**Authors:** Elisabeth Lipka, Sabine Müller

**Affiliations:** ZMBP, Developmental Genetics, University of Tübingen, Auf der Morgenstelle 32, D-72076 Tübingen, Germany

**Keywords:** Cytoskeleton dynamics, microtubule, nitrosative stress, phragmoplast.

## Abstract

Microtubules (MTs) are essential components of the cytoskeleton and fulfil multiple cellular functions in developmental processes, readily responding to intrinsic and external cues. Nitric oxide signalling is well established in plants, and the MT cytoskeleton is one of its potential targets. To mimic low level nitrosative stress, growth medium was supplemented with 3-nitro-l-tyrosine (NO_2_-Tyr), a nitrated form of the amino acid tyrosine, and concentration-dependent changes in root growth rate and a reduction in cell division frequencies in *Arabidopsis thaliana* were observed. In addition, it is reported that exposure to low NO_2_-Tyr concentrations was not detrimental to plant health and caused subtle and reversible defects. In contrast, growth defects caused by high NO_2_-Tyr concentrations could not be reversed. Live cell imaging of an MT reporter line revealed that treatment with a low concentration of NO_2_-Tyr correlated with disorganized cortical MT arrays and associated non-polar cell expansion in the elongation zone. NO_2_-Tyr treatment antagonized the effects of taxol and oryzalin, further supporting the association of NO_2_-Tyr with MTs. Furthermore, oblique division plane orientations were observed which were probably induced prior to cytokinesis.

## Summary

Mimicking low level nitrosative stress by supplementing seedlings with nitrated tyrosine induced subtle, reversible changes in root growth and in microtubule organization, suggesting that nitric oxid signalling targets the cytoskeleton.

## Introduction

Nitric oxide (NO) and its reactive derivatives act as intra- and intercellular signalling molecules in a variety of organisms (Besson-Bard *et al.*, 2008). In plants, NO is a key molecule triggering signalling cascades during development and in response to abiotic stresses (Neill *et al.*, 2002, 2008; He *et al.*, 2005; Lombardo *et al.*, 2006; Lombardo and Lamattina, 2012; León *et al.*, 2014). The NO concentrations for signalling during plant development are low, whereas high levels of NO induce programmed cell death (PCD) (Bai *et al.*, 2012). NO signalling acts upon diverse cellular responses including changes in gene expression levels via mitogen-activated protein kinase (MAPK) signalling cascades and regulation of transcription factors, as well as direct post-translational protein modifications (Neill *et al.*, 2002). The nitrated amino acid tyrosine (N-Tyr) is a highly specific product of NO signalling, although post-translational nitration does not affect all proteins to the same extent (Abello *et al.*, 2009). The impacts of N-Tyr incorporation into proteins include changes in enzymatic activity, alterations in proteolytic degradation, effects on protein phosphorylation and immunogenicity, and implications in disease (Abello *et al.*, 2009).

The cytoskeleton is a common downstream target of multiple signalling pathways leading to its extensive reorganization and consequent changes in cell morphology. Tubulin isoforms and many microtubule- (MT) associated proteins (MAPs) are conserved throughout the plant and animal kingdom. Increasing evidence in the literature suggests that the cytoskeleton might be a direct target of NO signalling in mammals as well as in plants. For instance, in mouse brain tissue, α- and β-tubulin isoforms were identified in a protein fraction enriched for N-Tyr (Zhang *et al.*, 2007). Similarly, α- and β-tubulin isoforms from *Arabidopsis thaliana* were immunopurified with anti-3nitroY antibody (Lozano-Juste *et al.*, 2011). Furthermore, N-Tyr-containing proteins were immunoprecipitated with anti-tubulin antibodies, demonstrating the incorporation of N-Tyr into tubulin isoforms (Yemets *et al.*, 2011). In mammalian cells, N-Tyr was incorporated into the extreme C-terminus of α-tubulin by tubulin–tyrosine-ligase (TTL), probably disturbing the tyrosination/detyrosination cycle of α-tubulin (Eiserich *et al.*, 1999). Substitution of the C-terminal tyrosine by N-Tyr correlated with MT disorganization and changes in cell morphology. The modification of the extreme C-terminus of α-tubulin by reversible enzymatically catalysed addition and removal of tyrosine is one of a number of well-characterized post-translational tubulin modifications (PTMs) in mammalian cells and plants, although in plants the respective enzymes have not been identified conclusively (Smertenko *et al.*, 1997; Westermann and Weber, 2003; Verhey and Gaertig, 2007; Giannoutsou *et al.*, 2012).

The significance of PTMs for MT functions, however, is not fully elucidated. Most PTMs occur at the C-terminal tail of tubulins and associate with subpopulations of MTs with distinct functions and subcellular localizations. Furthermore, immunolocalization of PTMs using a variety of PTM-specific antibodies revealed that combinations of different PTMs were present on individual MTs in mammalian and tobacco cells (Smertenko *et al.*, 1997; Janke and Kneussel, 2010; Quinones *et al.*, 2011). Thus, it was suggested that different PTMs might serve as road maps to ensure accurate cargo delivery (Verhey and Hammond, 2009). MAPs and, more specifically, kinesins emerged as possible targets of tyrosine signalling and PTM sensing (Gurland and Gundersen, 1995).

In plants, the MT cytoskeleton enables diverse cellular functions and is an integral constituent of developmental processes. In directionally expanding interphase cells cortical MTs arrange in parallel bundles, transverse to the axis of expansion, and guide plasma membrane-resident cellulose synthase complexes (Chan *et al.*, 2007; Gutierrez *et al.*, 2009). In mitotically active cells of higher plants, the cytoskeletal pre-prophase band is indicative of the future site of cell division. During cytokinesis, the phragmoplast facilitates the synthesis and fusion of the cell plate at the site formerly occupied by the pre-prophase band (Müller, 2012; Rasmussen *et al.*, 2013).

Recently it has been proposed that N-tyrosinylation of the α-tubulin C-terminus might play a role in plant cell division (Jovanović *et al.*, 2010). Rice seedlings and tobacco suspension culture cells were grown in the presence of 3-nitro-l-tyrosine (NO_2_-Tyr) as an exogenous source of N-Tyr, to add N-Tyr irreversibly to the C-terminus of α-tubulins. Upon treatment, tobacco cell culture cells exhibited mitotic inhibition and obliquely orientated cross walls, supporting the idea of direct impact of NO_2_-Tyr on prominent MT functions. In a recent report, growth defects of NO_2_-Tyr were investigated in *A. thaliana* (Blume *et al.*, 2013). Here growth defects and associated MT reorganization in *A. thaliana* seedlings grown in the presence of NO_2_-Tyr are reported. Changes in MT organization were directly monitored, using live cell imaging of MTs, and visualized by a fluorescent MT reporter. In a concentration-dependent manner, NO_2_-Tyr decreased mitotic activity and caused cell swelling due to re-organized cortical MT arrays; however, the effects of oryzalin or taxol were less pronounced in combination with NO_2_-Tyr. Thus, *in vivo* evidence is provided that modulation of NO signalling allows for the reorganization of the MT cytoskeleton that might be relevant for development.

## Materials and methods

### Plant material and growth conditions

Throughout this study, transgenic *A. thaliana* plants, accession Columbia (Col) and Col expressing green fluorescent protein (GFP)–MAP4 (Marc *et al.*, 1998), were used unless indicated otherwise. Seeds were surface sterilized, plated on standard medium plates containing 1× Murashige and Skoog salt mixture (pH 5.7) and 1% agar, and were stratified for 2–4 d at 4 °C. In the growth chamber, plates were positioned vertically and seedlings grew for 4 d at 22 °C with continuous light (standard conditions). Under sterile conditions, seedlings were then transferred onto nutrient agar plates containing different concentrations of NO_2_-Tyr (Sigma-Aldrich). Treatment plates were prepared from standard medium supplemented with varying amounts of NO_2_-Tyr from 1mM NO_2_-Tyr stock solution dissolved in 50 μM HCl or dimethylsulphoxide (DMSO). Mock controls contained 0.5 μM HCl and 10 μM tyrosine.

### Construction of XFP–fusion proteins

TUA6 cDNA was synthesized as described elsewhere (Muller *et al.*, 2006). Primers TUA6 F 5′-*GGTACC*ATGAGAGAGTGCATTTCGATCCA-3′ and TUA6 R 5′-*CTCGAG*TTAGTATTCCTCTCCTTCATCAT-3′ were used to amplify the TUA6 coding sequence flanked by *Kpn*I and *Xho*I restriction sites (indicated in italics). Subse quently, cTUA6 was cloned into pGEM T (Promega), sequence verified, and transferred into pENTR3C via cloning into the *Kpn*I and *Xho*I sites. Primers TUA6-Y224A-F 5′-AACATTGAGAGACCTACC**GCC**ACCAA-3′ and TUA6- Y224_R 5′-TTGAGGTTGGT**GGC**GGTAGGTCTCTCAAT-3′ were used for site-directed mutagenesis to create pENTR-cTUA6 Y224A. To create pENTR cTUA6 Y450A, primer TUA6 F was used with TUA6-Y450A R 5′-*CTCGAG*TTA**GGC**TTCCTC TCCTTCATCAT-3′ for amplification of a PCR fragment which was digested with restriction enzymes *Eco*RI (TU6 internal site) and *Xho*I to exchange the respective fragment in pENTR cTUA6. Finally, pENTR clones were recombined with pFK-241 pGreenIIS destination vector using LR clonase (Invitrogen).

The MT-binding domain (MBD) contained in the destination vector pEG104-*35S*::mCherry-MBD (Gutierrez *et al.*, 2009) was recombined into pDONR207 by BP clonase reaction (Invitrogen). Subsequently, an LR clonase reaction (Invitrogen) was performed with the destination vector *pUBN*:RFP containing the *Arabidopsis* ubiquitin10 promoter (*pUB*) (Grefen *et al.*, 2010).

### Imaging and data analysis

Microscopic images were acquired on a Leica SP2 upright confocal microscope using either a ×20, NA=0.70 water-immersion objective lens or a ×63, NA=1.20, water-immersion objective lens. Imaging of mitotic MT arrays was performed on a Leica SP8 inverted confocal microscope, with a ×40, NA=1.10, water-immersion objective lens, equipped with a resonant scanner. GFP was excited with the 488nm emitting line of argon lasers. Cell wall patterns were visualized by staining with propidium iodide (10 µg/ml, Sigma-Aldrich) which was excited with a 561nm HeNe laser. Images were acquired using Leica AF software. Image analysis and processing was performed in ImageJ (http://rsbweb.nih.gov/ij/) and Adobe Photoshop CS5, respectively. Figures were assembled in Adobe Illustrator CS5.

The density of MT bundles per micrometre was determined as described previously in Tanou *et al.* (2012). In brief, z-projections were assembled in ImageJ and grey values of Plot Profiles were measured and background corrected by subtracting the mean grey values of cortex areas without MTs. Along the plot profile, peaks were defined as grey values which were at least 10 units higher than the grey values of both neighbouring pixels, using Excel. Six intensity classes were deduced and the numbers of peaks were counted for each intensity class. Peaks are indicative of MT bundles.

The intensity of the cytosolic GFP–MBD signal in root cells of the elongation zone was determined in maximum projections of 21 optical sections at 0.5 μm intervals. Fluorescence intensities of areas between cortical MTs, corresponding to unbound cytosolic GFP–MBD, were measured. Averaged grey values per cell were background-substracted and plotted in Excel.

The Kymograph Plugin (http://www.embl.de/eamnet/html/body_kymograph.html) was used to determine MT plus-end dynamicity, and measurements were used to calculate average growth and shortening velocities in Excel. Pearsons correlation coefficient was calculated using the Colocalization Finder Plugin in ImageJ (http://rsb.info.nih.gov/ij/plugins/colocalization-finder.html).

Statistical significance of differences in measurements was determined with Student’s *t*-test.

## Results

### Effects of nitro-tyrosine on root growth

To mimic nitrosative stress conditions *A. thaliana* seedlings were exposed to different concentrations of 3-nitro-l-tyrosine [designated NO_2_-Tyr to distinguish it from intracellular/endogenous (N-Tyr) concentrations] added to the growth medium. Then, the effects of N-Tyr imbalance on seedling development were assessed. Therefore, 4-day-old seedlings expressing the MT reporter GFP–MBD were transferred to growth media containing varying concentrations of NO_2_-Tyr (0.1, 0.25, 0.5, 1, 5, and 10 μM) and grown for an additional 36h (1.5 d). Control seedlings were transferred to standard medium, or medium containing either 0.5 μM HCl, 10 μM Tyr, or both (mock controls).

**Fig. 1.**
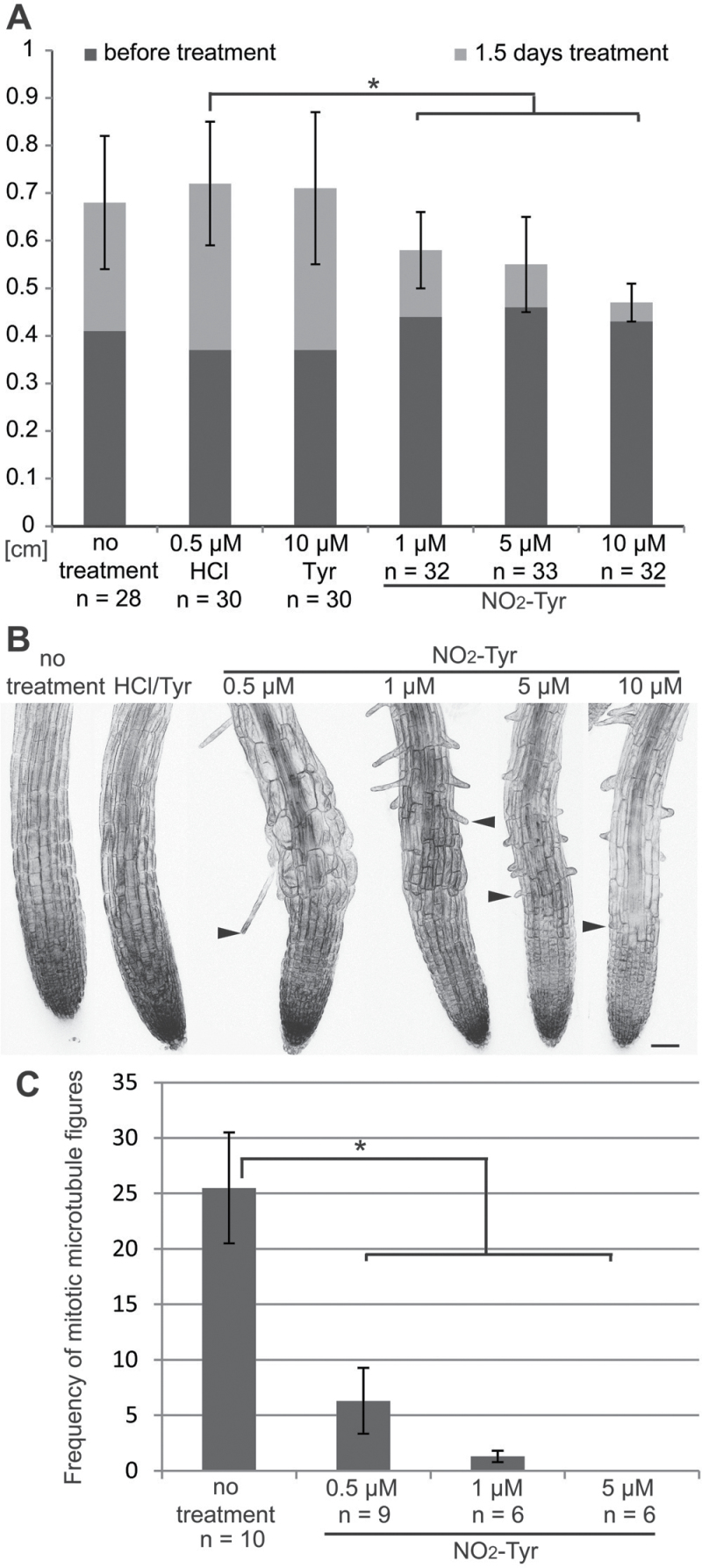
*Arabidopsis thaliana* seedling root growth and root tip morphology. (A) Average root length of seedlings at 4 d after germination (dark grey bar) and subsequently after 1.5 d of exposure to different amounts of 3-nitro-l-tyrosine (NO_2_-Tyr) in the growth medium (light grey bar). Root growth during the 1.5 d treatment (light grey bars) is significantly diminished on NO_2_-Tyr-containing medium (1 μM NO_2_-Tyr 0.14±0.08cm, 5 μM NO_2_-Tyr 0.09±0.01cm, 10 μM NO_2-_Tyr 0.04±0.04cm, *P≤0.015) compared with controls, which show insignificant differences in root growth (no treatment 0.27±0.14cm, 0.5 μM HCl 0.35±0.13cm, 0.34±0.16cm, *P*≥0.21). Sample sizes (*n*) for each treatment are given. Error bars indicate ±standard deviation (SD). (B) Confocal images of seedling root tips expressing the microtubule reporter GFP–MBD. Images are maximum intensity z-projections of image stacks taken at 10 μm intervals; the number of images per stack varies. Seedlings grown under control conditions (10 μM Tyr, 0.5 μM HCl) did not exhibit changes in root morphology. Seedlings exposed to NO_2_-Tyr displayed moderate cell expansion defects in the root elongation zone (0.5 μM and 1 μM NO_2_-Tyr) and short root hairs (1, 5, and 10 μM NO_2_-Tyr) as indicated by arrowheads. Scale bar=50 μm. (C) Exposure of seedlings to different amounts of NO_2_-Tyr inhibits mitotic activity in the root meristems. The frequency of mitotic microtubule arrays (pre-prophase band, spindle, and phragmoplast) decreased significantly (*P<0.01) in NO_2_-Tyr-treated seedlings. The numbers (*n*) of analysed root meristems are indicated for each individual treatment. Bars represent mean quantities of mitotic microtubule arrays. Error bars indicate ±SD. (This figure is available in colour at *JXB* online.)

Analysis of root length before and after treatment revealed a dramatic reduction in growth upon NO_2_-Tyr exposure compared with the non-treated and the mock control seedlings (Fig. 1A). The reduction in root length was significant and decreased further with increasing NO_2_-Tyr concentrations (Fig. 1A). In contrast, root growth of untreated controls and mock-treated seedlings (0.5 μM HCl, 10 μM Tyr) did not vary (Fig. 1A; Supplementary Fig. S2A available at *JXB* online). In addition, the use of either HCl or DMSO as the solvent did not make a difference in growth responses (Supplementary Fig. S2B). Also, upon NO_2_-Tyr treatment, the growth response of wild-type plants (Col) and GFP–MBD transgene plants was comparable (Supplementary Fig. S3A, B).

Seedlings grown on NO_2_-Tyr exhibited altered tip organization. At low concentrations of NO_2_-Tyr (0.5 μM and 1 μM), cells in the elongation zone displayed non-polar expansion (Fig. 1B). Intriguingly, high concentrations (5 μM and 10 μM) did not induce morphological alterations, suggesting that high amounts of NO_2_-Tyr rapidly interfered with cellular functions. Moreover, a concentration-dependent reduction of the meristem and elongation zone was apparent between the root tip and the first root hair (Fig. 1B; Supplementary Fig. S1A at *JXB* online). In addition, the elongation of root hairs was affected at NO_2_-Tyr concentrations ≥1 μM (Fig. 1B).

### Mitosis is inhibited in nitro-tyrosine-treated seedlings

Observations on rice seedlings and tobacco Bright Yellow (BY)-2 suspension culture cells suggested that NO_2_-Tyr treatment inhibited cell division (Jovanović *et al.*, 2010). Expression of the MT reporter GFP–MBD allowed the evaluation of mitotic activity in living *A. thaliana* seedlings (Supplementary Fig. S1B at *JXB* online). The number of mitotic cells between the quiescent centre and epidermis was counted in image stacks at 2 μm z-intervals (Supplementary Fig. S1B). In control plants, on average 25.5 mitotic MT structures were observed per image stack (Fig. 1C; Supplementary Fig. S1B). In contrast, equivalent image stacks of NO_2_-Tyr-treated seedlings revealed a significant reduction in mitotic activity. At 0.5 μM NO_2_-Tyr the mean number of mitotic MT structures dropped to 6.1 and at a concentration of 1 μM NO_2_-Tyr only 1.3 mitotic cells were observed on average. Mitotic activity was entirely inhibited at 5 μM NO_2_-Tyr. These results confirmed a concentration-dependent inhibition of cell divisions upon NO_2_-Tyr treatment in *A. thaliana* seedlings.

### NO_2_-Tyr-induced growth inhibition effects are reversible at low concentrations

**Fig. 2.**
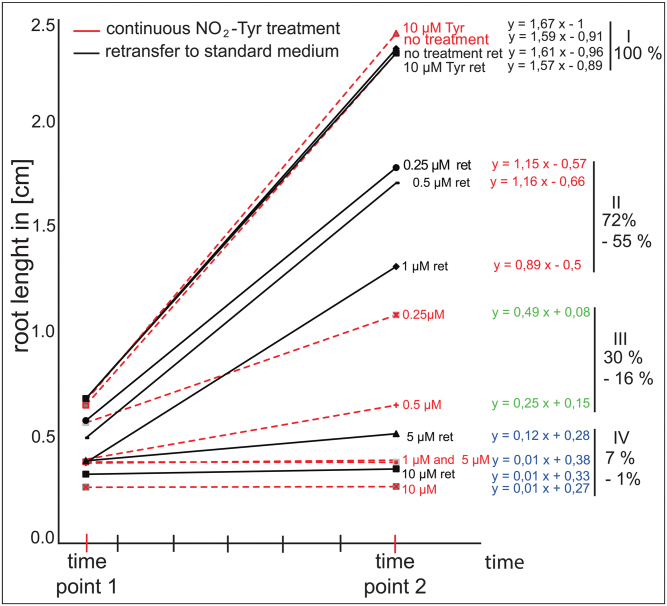
Growth effects induced by 3-nitro-l-tyrosine (NO_2_-Tyr) concentrations are reversible. Seedlings were grown on standard medium for 4 d and were subsequently transferred to growth medium containing different NO_2_-Tyr concentrations as indicated for 1.5 d (time point 1). Then, half of the seedlings were retransferred to standard medium for an additional 4.5 d (time point 2). Root length before (time point 1) and 4.5 d after retransfer (time point 2) is plotted over time. Black curves (continuous line) indicate samples retransferred onto standard medium plates, and red curves (dashed lines) indicate samples that were transferred to freshly prepared NO_2_-Tyr medium (continuous NO_2_-Tyr treatment). Equations of linear regression curves are indicative of growth rate performance and are given for each treatment. The equations are grouped into four classes (I –IV) based on the similarity of their slope. Class I contains non-transferred and retransferred control samples and represent 100% of the growth rate potential. Class II comprises samples retransferred from low NO_2_-Tyr concentrations (0.25, 0.5, and 1 μm ret). Class III contains samples continuously treated with low NO_2_-Tyr concentrations (0.25 μM and 0.5 μM). Class IV includes high NO_2_-Tyr concentrations (1, 5, and 10 μM, 5 μM ret and 10 μM ret). Percentage ranges indicate growth performance as achieved in each class as a means to illustrate growth recovery relative to the potential growth as measured for controls (100%).

Since high concentrations of NO_2_-Tyr had dramatic effects on root growth, it was investigated whether low concentrations or extended treatment periods were harming cellular functions to a similar extent and whether NO_2_-Tyr-induced effects on root growth could be reversed. As described above, seedlings were grown under standard conditions for 4 d and were subsequently transferred onto NO_2_-Tyr-containing medium for an additional 1.5 d. Then, the root length was measured, and half of the seedlings were retransferred onto standard medium for an extra 4.5 d (Fig. 2, black lines, designated as ‘ret’ for retransfer) to test whether root growth defects were reversed. The remaining half of the seedlings was transferred onto freshly prepared treatment plates of the respective NO_2_-Tyr concentration (Fig. 2, red lines indicating continuous NO_2_-Tyr treatment).

The root length of seedlings under different treatment conditions was plotted over time, and regression equations for each treatment were calculated as a reference for growth performance (Fig. 2). Based on similarities of the regression equations, the samples were grouped into four classes (I–IV). Controls (class I) were set to 100%, representing the full growth potential. Class II included low NO_2_-Tyr samples that were retransferred to standard medium (0.25 μM ret and 0.5 μM ret), attaining 72% recovery of potential growth. In addition, the 1 μM ret sample was grouped with class II, still displaying 55% of the potential growth. In contrast, samples that continuously grew on low NO_2_-Tyr concentrations (0.25 μM and 0.5 μM) fell into class III possessing only between 30% and 16% of control growth potential. Finally, class IV comprised all high concentration samples (5 μM and 10 μM continuous NO_2_-Tyr and retransferred 5 μM ret and 10 μM ret), all of which displayed severely reduced growth (1% growth). In addition, class IV also included samples continuously grown on 1 μM NO_2_-Tyr (7% growth). The clustering of samples into distinct groups indicated that high concentrations of NO_2_-Tyr irreversibly harmed seedlings even during the initial short-term treatment (1.5 d). On the other hand, low NO_2_-Tyr concentrations caused mild, non-toxic effects which were reversible during the same treatment period. Remarkably, continuous treatment with 0.25 μM NO_2_-Tyr still allowed 30% growth, while short-term treatment with 1 μM NO_2_-Tyr could still be reversed to about half (55%) of the potential growth.

### Cortical microtubule arrays are disordered upon NO_2_-Tyr treatment

**Fig. 3.**
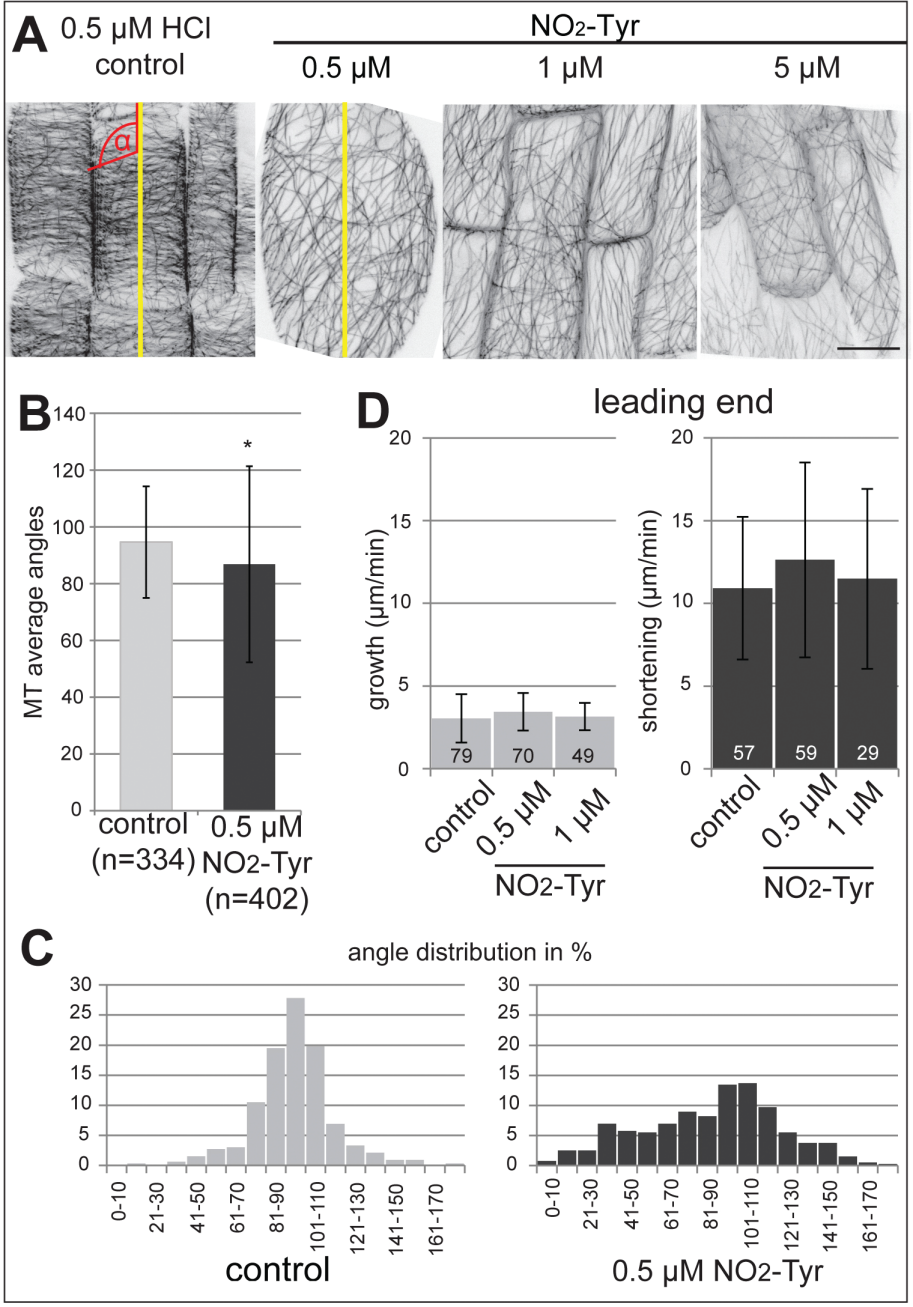
Organization of cortical microtubule (MT) arrays in epidermal cells at the elongation zone. (A) In controls, interphase cells show characteristic parallel alignment of cortical MTs. Low concentrations of 3-nitro-l-tyrosine (NO_2_-Tyr, 0.5 μM) disrupt the cortical MT array organization and cells show signs of non-polarized expansion. Representative, inverted images are maximum intensity z-projections of image stacks, taken at 1 μm z-intervals; stacks encompass optical sections from the surface to a median cross-section through the cells. The number of optical sections per stack varies. Scale bar=10 μm. (B–D) Distribution of MT angles and the dynamicity of MT plus (leading) ends in epidermal cells of the elongation zone; (B) MT angles (red angle symbol in A) were determined counter clockwise, relative to the long axis of the cell (indicated by the yellow lines in A). The average angle of MTs (86.8±34.6 °) in NO_2_-Tyr-treated cells differs significantly from the average angle in control cells (94.6±19.6 °). The total number (*n*) of evaluated MT angles is given. Results represent the average angle of *n*=8 cells calculated from eight plants for controls and the average angle calculated from *n*=7 cells of seven plants treated with 0.5 μM NO_2_-Tyr. The average angle of cortical MTs upon NO_2_-Tyr treatment is significantly different from the transverse angle observed in controls (*P=0.0003). It is noteworthy that the standard deviation is rather large for NO_2_-Tyr. (C) Distribution histogram of MT angles observed in relation to the long axis of the cell in (A); the *x*-axis represents MT angles in 10 ° bins and the *y*-axis depicts the frequency of measurements for each bin. (D) Growth (polymerization) and shortening (depolymerization) velocities were determined for MT plus (leading) ends. No significant differences in MT dynamicity were observed between no treatment controls and NO_2_-Tyr-treated cells. MT growth and shortening velocities were measured in controls (*n*=23 cells from 10 roots), 0.5 μM NO_2_-Tyr-treated (*n*=28 cells from 11 roots), and 1 μM NO_2_-Tyr-treated (*n*=21 cells from eight roots) plants. The numbers of analysed plus ends are indicated in the bar diagram. Results represent the average velocities of two independent experiments. Error bars indicate ±standard deviation.

Low concentrations of NO_2_-Tyr (0.5 μM) caused non-polar cell expansion in the elongation zone after 1.5 d of treatment, while cell division and cell expansion ceased at concentrations >1 μM NO_2_-Tyr (Fig. 1B, C). Non-polar cell expansion is typically related to alterations in MT organization. Indeed, in NO_2_-Tyr-treated plants, the characteristic parallel alignment of the cortical MT array was disturbed in cells with obvious expansion defects (Figs 1B, 3A). Rather, cortical MT arrays were disordered and appeared less dense than in control cells (Fig. 3A). Intensity-based quantification of MT bundles along the long axis of cells (as indicated by the yellow lines in Fig. 3A and corresponding blot profiles in Supplementary Fig. S4A at *JXB* online) revealed an overall reduction of thick and thin MT bundles in 0.5 μM NO_2_-Tyr-treated cells (Supplementary Fig. S4B). The average number of MT bundles per micrometre was significantly smaller in treated cells (1.05±0.12, *P<0.05) compared with control cells (1.32±0.18, Supplementary Fig. S4C). Consistent with the reduction of thick and thin MT bundles, the intensities of cytosolic GFP–MBD fluorescence increased upon increasing concentration of NO_2_-Tyr (Supplementary Fig. S4D). To assess the degree of disorder, angles between MT bundles and the cell long axis (as depicted in Fig. 3A, red angle symbol) were measured. In cells of treated seedlings, the mean direction of MT bundles (86.8±34.6 °) deviated significantly (*P=0.0003) from the mean MT angle calculated for control cells (94.6±19.6 °). Moreover, the high standard deviation indicated a larger variability in the data set for NO_2_-Tyr-treated cells (Fig. 3B) which became more obvious when the frequencies of angles were blotted in 10 ° bins (Fig. 3C). In controls the majority of MT angles (67.1%) ranged between 80 ° and 110 °. However, upon NO_2_-Tyr treatment, only 35.5% of cortical MTs displayed angles between 80 ° and 110 °, while the number of greater and smaller angles increased. It is noteworthy that the distribution across the 10 ° bins in the control resembled a normal distribution curve with a distinct peak, while the angles in NO_2_-Tyr treatment displayed a more homogeneous, flat distribution across the degree bins. In reversibility experiments, parallel MT array organization was restored at low NO_2_-Tyr concentrations (data not shown).

In order to determine whether loss of cortical MT organization was caused by altered MT dynamicity, growth velocities (indicative of MT polymerization) and shortening velocities of MT plus ends (indicative of MT depolymerization) were measured in epidermal cells of the elongation zone. Interestingly, no significant differences were observed between growth velocities in control (3.04±1.46 μm min^–1^), 0.5 μM NO_2_-Tyr-treated (3.44±1.14 μm min^–1^), and 1 μM NO_2_-Tyr-treated seedlings (3.15±0.82 μm min^–1^) or for shortening velocities in control (10.91±4.31 μm min^–1^), 0.5 μM NO_2_-Tyr-treated (12.63±5.89 μm min^–1^), and 1 μM NO_2_-Tyr-treated seedlings (11.48±5.43 μm min^–1^) (Fig. 3D).

### Low concentrations of NO_2_-Tyr counteract responses to taxol and oryzalin

Both oryzalin and taxol stimulate non-polar cell expansion through their interaction with tubulin (Baskin *et al.*, 1994). By combining 0.5 μM NO_2_-Tyr with different concentrations of either taxol or oryzalin, changes were observed in the growth rate and MT organization in seedlings exposed to the drug treatment, as described before.

**Fig. 4.**
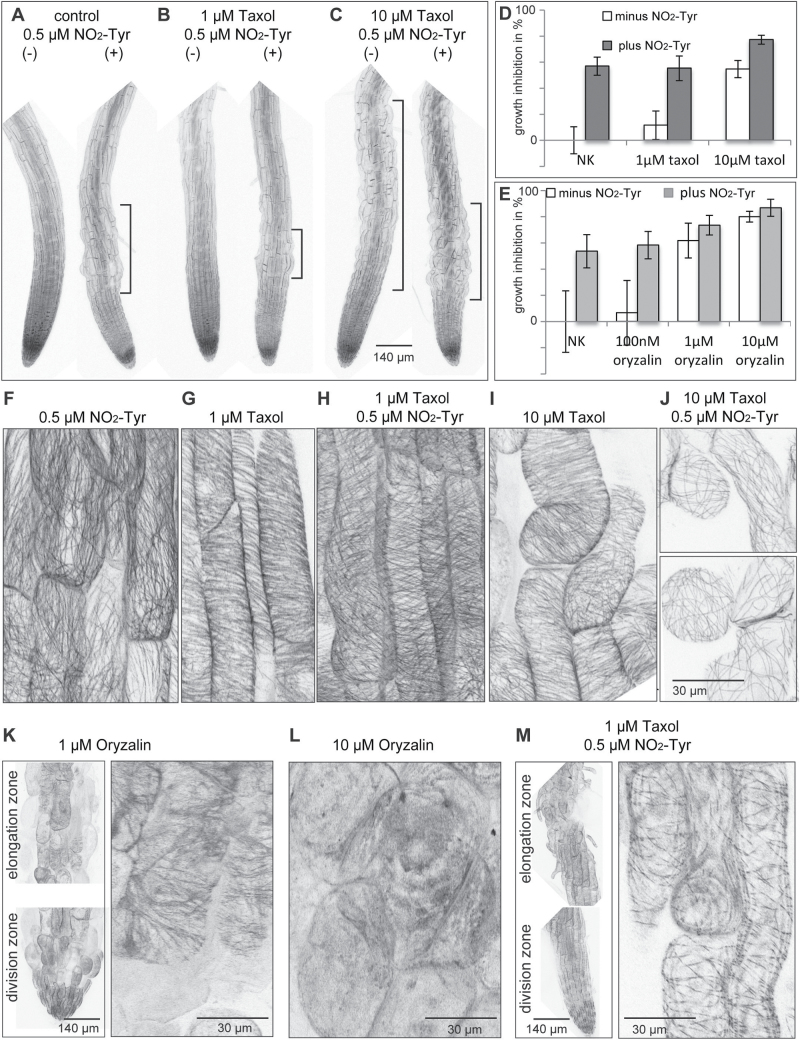
Effects of NO_2_-Tyr in combination with taxol or oryzalin. (A–C) Seedling morphology of (A) control without (–) and with (+) 0.5 μM NO_2_-Tyr. (B and C) Taxol treatment as indicated without (–) and with (+) 0.5 μM NO_2_-Tyr. (D and E) Growth inhibition is displayed as a percentage of untreated controls. (D) White columns show growth inhibition with taxol only. Grey columns show growth inhibition in the presence of 0.5 μM NO_2_-Tyr. Error bars indicate ±standard deviation (*n*≥24). (E) Normalized growth inhibition as a percentage of control. White columes show growth inhibition with oryzalin only. Grey columes show growth inhibition in the presence of 0.5 μM NO_2_-Tyr. Error bars indicate ±standard deviation (*n*≥27). (F–M) Microtubule (MT) organization in the presence of drugs as indicated. (K and M) Left panel: morphology of the elongation zone and division zone upon drug treatment as indicated.

Taxol alone had little (11% for 1 μM taxol) or similar effects (54% for 10 μM taxol) on growth inhibition (as a percentage of control) compared with 0.5 μM NO_2_-Tyr alone (56%) (Fig. 4A, D). Growth inhibition by taxol in combination with NO_2_-Tyr (55% and 77%) was mostly attributed to NO_2_-Tyr effects (Fig. 4B–D). At the cellular level, taxol-induced stabilization of MTs (Fig 4G, 4I) was counteracted by NO_2_-Tyr when both drugs were present in the growth medium (Fig. 4H). High levels of taxol combined with NO_2_-Tyr caused pronounced cell expansion defects and NO_2_-Tyr-induced re-organization of MTs (Fig. 4J), indicating that the effects of taxol on MTs were over-ridden by NO_2_-Tyr.

**Fig. 5.**
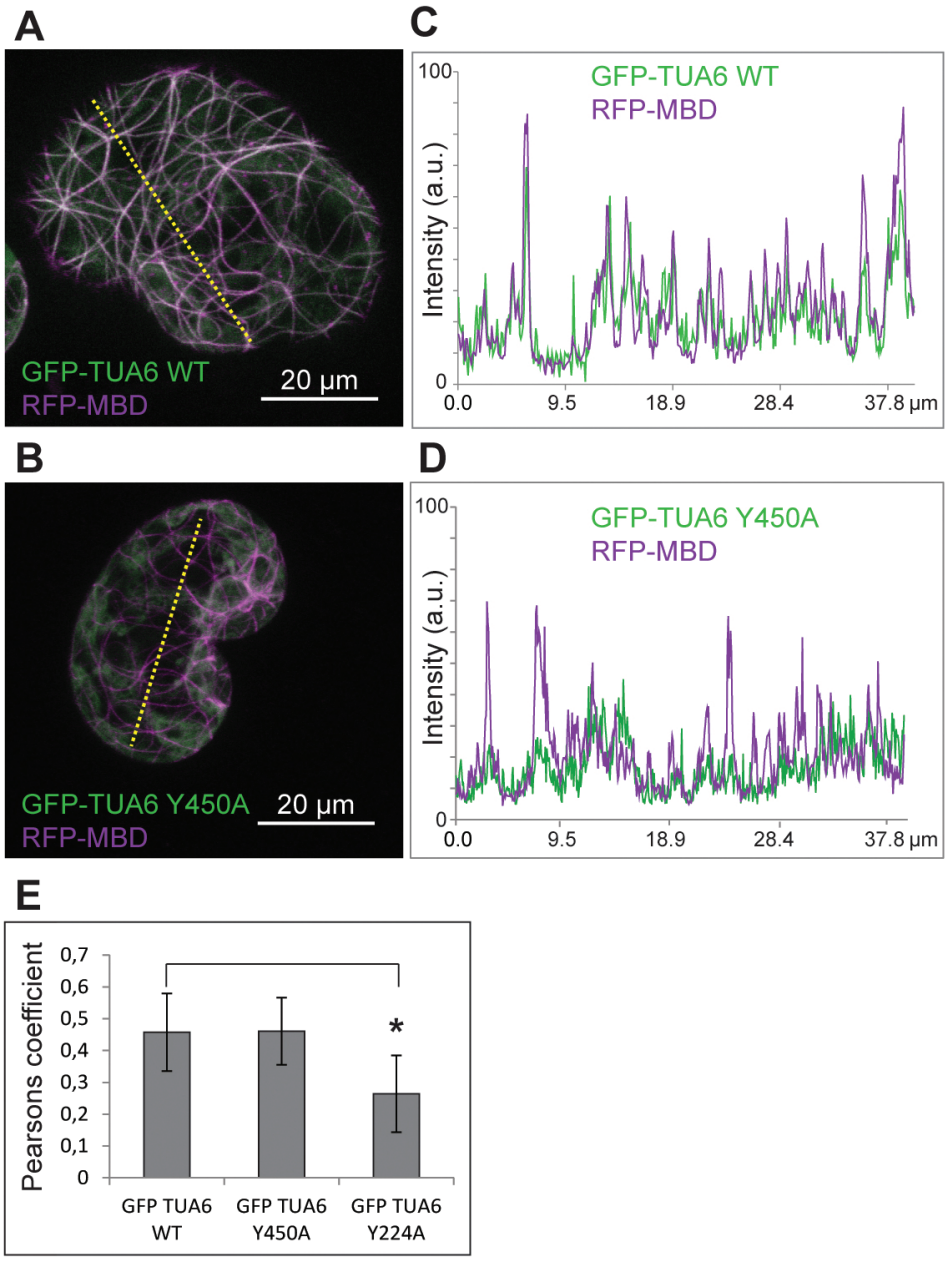
*Arabidopsis thaliana* protoplasts co-expressing TUA6 *p35S:GFP-TUA6* (green) and *p35S:RFP-MBD* (magenta). (A) Protoplast co-expressing *p35S:GFP-TUA6 WT* and *p35S: RFP-MBD*. (C) Plot profile of fluorescent signal intensities along the dotted line in (A). (B) Protoplasts co-expressing *p35S:GFP-TUA6 Y450A* and *p35S: RFP-MBD*. (D) Plot profile of fluorescent signal intensities along the dotted line in (B). (E) Mean Pearsons correlation coefficient determined for GFP and RFP in protoplasts co-expressing *p35S:RFP-MBD* together with either *p35:GFP-TUA6 WT* (0.45±0.12, *n*=14) or mutant *p35:GFP-TUA6 Y450A* (0.46±0.10, *n*=14) and *p35:GFP-TUA6 Y224A* (0.26±0.12, *n*=14, *P>0.001).

Oryzalin treatment disrupted MTs, causing irregular expansion of cells and inhibition of mitosis, both contributing to a concentration-dependent reduction in growth rate (Fig. 4E, K, L). The effects of 1 μM oryzalin on growth inhibition (74%) were more severe than those of 0.5 μM NO_2_-Tyr alone (65%) (Fig. 4E). However, in combination with 0.5 μM NO_2_-Tyr, the oryzalin effect on growth rate inhibition increased only to 78% (Fig. 4E, M). Consistently, the addition of 0.5 μM NO_2_-Tyr reduced the oryzalin-dependent destabilization of MTs (Fig. 4M), suggesting that oryzalin binding to tubulin might be less efficient in the presence of NO_2_-Tyr. Single mutations in helix 7 (H7) of α-tubulins were reported to render plants and protozoa resistant to oryzalin (Anthony *et al.*, 1998; Morrisette *et al.*, 2004; Supplementary Fig. S5 at *JXB* online). Therefore, tyrosine at the beginning of H7 was mutated to alanine (Y224A) in TUA6 (Fig. 5A), which was identified among nitrotyrosinated proteins in *A. thaliana* (Lozano-Justo *et al.*, 2011; Supplementary Fig. S5A), and it was expressed as a GFP fusion in *A. thaliana* protoplasts. Similarly, the C-terminal tyrosine in TUA6, the putative target of the tyrosination/detyrosination cycle, was mutated to alanine (Y450A) (Fig. 5A). Wild-type TUA6 (TUA6 WT) and mutant GFP fusion proteins were co-expressed with red fluorescent protein (RFP)–MBD to visualize MTs (Fig. 5B, D). While TUA6 WT (Fig. 5B, C, F) and the TUA6 Y450A mutant showed co-localization with RFP–MBD (Fig. 5F), TUA6 Y224A was not efficiently incorporated into MTs as determined by Pearsons correlation coefficient (Fig. 5D–F), supporting a critical role for Y224 for the integrity of the tubulin molecule.

### Long-term, low concentration NO_2_-Tyr treatment affects division plane orientation in root meristems

**Fig. 6.**
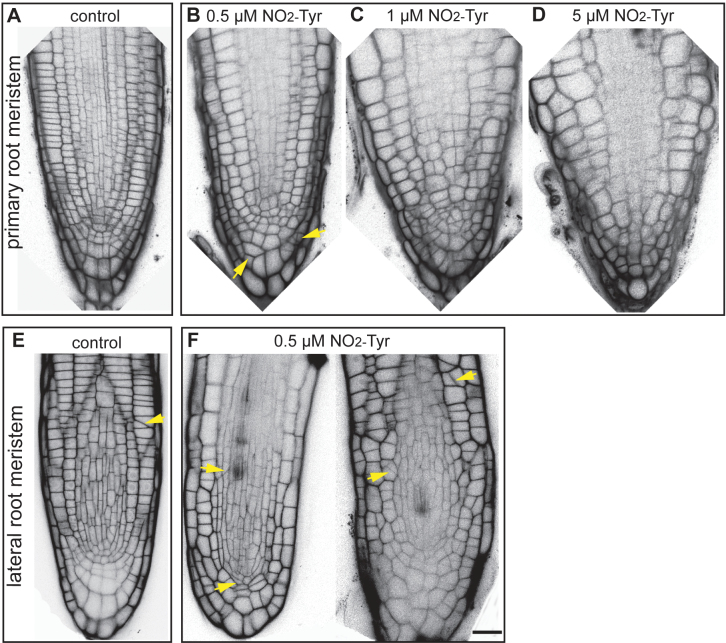
Long-term treatment at low 3-nitro-l-tyrosine (NO_2_-Tyr) concentrations moderately affects division plane orientation in root meristems. Confocal images show cell wall patterns of root meristems stained with propidium iodide. (A and E) No treatment controls display regular stereotypic cell division patterns. (B, C, F) Low amounts of NO_2_-Tyr (0.5 μM and 1 μM) have modest effects on cell wall positioning, exhibiting a low frequency of oblique cell walls (arrows). (D) No oblique walls were observed in 5 μM NO_2_-Tyr-treated meristems due to the inhibition of mitotic activity at high NO_2_-Tyr concentrations.

In BY-2 cells, NO_2_-Tyr treatment altered the division planes (Jovanović *et al.*, 2010). It was decided to determine whether similar defects occurred in the tissue context of *A. thaliana* root meristems. In short-term (1.5 d) experiments, cell wall positioning was normal; however, under high NO_2_-Tyr concentrations (1–10 μM), mitosis was almost abolished and was already reduced at 0.5 μM NO_2_-Tyr (Fig. 1C; Supplementary Fig. S1B). Therefore, cell wall positions in the root meristem were visualized by propidium iodide staining and were analysed in seedlings exposed to 0.5 μM NO_2_-Tyr for 6 d. It was reasoned that cell divisions still occurred at this concentration (Fig. 1C) and thus might permit the accumulation of cell wall positioning defects over time. Indeed, a low number of oblique cell walls were observed in primary (Fig. 6B) and lateral root meristems (Fig. 6F) compared with the wild type (Fig. 6A, E).

However, positioning defects in NO_2_-Tyr-treated plants were modest compared with mutants exhibiting defects in this pathway, such as the double mutant of *PHRAGMOPLAST ORIENTING KINESIN 1* and *2* (*POK1* and *2*; Muller *et al.*, 2006). While *pok* single mutants are phenotypically wild type, *pok1pok2* double mutants display severely misplaced cell walls due to a failure in phragmoplast guidance (Muller *et al.*, 2006). Thus, it was investigated whether *pok1* and *pok2* single and *pok1pok2* double mutants were hypersensitive to NO_2_-Tyr and whether cell wall positioning defects in these plants were induced at lower NO_2_-Tyr concentrations than in GFP–MBD plants. As in previous experiments, seedlings were exposed to different NO_2_-Tyr concentrations and the growth performance was assessed. Both *pok1pok2* double mutants and *pok1* single mutants, as well as *pok2* single mutants expressing GFP–MBD (*pok2;*GFP–MBD), showed NO_2_-Tyr-induced growth reduction similar to GFP–MBD transgenic plants (Supplementary Fig. S4 at *JXB* online). Furthermore, cell wall positioning defects were comparable with those of wild-type plants (data not shown). Thus it was concluded that NO_2_-Tyr had no effect on either *pok1*, *pok2;*GFP–MBD, or *pok1pok2* double mutants (Supplementary Fig. S6) and that NO_2_-Tyr-induced oblique cell walls were not a result of interference with phragmoplast guidance.

## Discussion

In all organisms investigated so far including plants, the abundance of NO and its derivatives such as free NO_2_-Tyr is regarded as a reporter of nitrosative stress under pathological conditions (Halliwell *et al.*, 1999; Foissner *et al.*, 2000; Besson-Bard *et al.*, 2008; Moreau *et al.*, 2010). In plants, biotic and abiotic stress conditions lead to NO imbalance and provoke nitrosative responses preceding physiological and developmental changes (Lombardo *et al.*, 2006; Neill *et al.*, 2008; Corpas *et al.*, 2011; Tanou *et al.*, 2012). A solid understanding of NO biosynthesis and the *in vivo* effects of NO signalling is emerging in the plant field (Besson-Bard *et al.*, 2008; Moreau *et al.*, 2010). However, the detection of low, physiologically and developmentally relevant concentrations of reactive nitrogen species remains challenging (Halliwell *et al.*, 1999; Berton *et al.*, 2012). In the present study, the impacts of different concentrations of exogenously supplied NO_2_-Tyr on root growth, root tip morphology, MT organization, and phragmoplast guidance in *A. thaliana* seedlings were evaluated.

A recent study in *A. thaliana* roots reported that NO stress, induced by sodium nitroprusside (SNP), caused PCD at high SNP levels, but triggered cell cycle arrest in G_1_ phase at medium SNP levels, which were still above the reported developmentally relevant concentrations (Bai *et al.*, 2012). Consistent with the present findings in response to different NO_2_-Tyr concentrations, root growth was reduced and meristem cell number decreased upon SNP treatment (Bai *et al.*, 2012). In the experiments reported here, the number of cell divisions decreased significantly at high NO_2_-Tyr concentrations, indicative of cell cycle arrest. Although parameters such as DNA damage were not analysed, it is plausible that DNA damage induced at high NO_2_-Tyr concentrations was responsible for the observed irreversible arrest of plant growth. The reversibility experiments are consistent with a recent study reporting reversibility of growth-inhibiting effects (Blume *et al.*, 2013) at NO_2_-Tyr concentrations similar to those used in this study. While stunted and moderately depolarized root hairs were observed here, 10-fold higher NO_2_-Tyr caused ectopic and distorted root hairs (Blume *et al.*,2013), reflecting the wide morphological impact of nitrosative stress.

NO is a developmentally important signalling molecule in plants (Moreau *et al.*, 2010). For instance, NO signalling targets root development via signal transduction to a MAPK signalling cascade (Wang *et al.*, 2010). NO is also a significant intermediate of abscisic acid-induced signalling in the control of stomatal aperture (He *et al.*, 2005; Ribeiro *et al.*, 2009). In this context, NO targets the protein phosphatase 2C ABI1, but whether NO directly modifies ABI1, as has been shown for H_2_O_2_, is unclear (Meinhard and Grill, 2001; Desikan *et al.*, 2002).

However, direct modification of cytoskeletal proteins might represent one disposition of NO signal propagation. In mammalian cells, N-Tyr is incorporated into the C-terminus of α-tubulin under pathological conditions, strongly suggesting that the cytoskeleton might be one target of NO signalling (Eiserich *et al.*, 1999). Moreover, in BY-2 cells, NO_2_-Tyr treatment correlated with a decrease in the relative amount of detyrosinated α-tubulin isoforms (Jovanović *et al.*, 2010).

Several recent studies have reported on cytoskeletal targets of NO signalling or nitrosative stress. In protein extracts from BY-2 cells, antibodies against NO_2_-Tyr preferentially decorated proteins of ~55kDa molecular weight, similar to anti-tubulin antibodies, and precipitated tubulin cross-reacted with anti-tubulin antibodies, in support of the proposed incorporation of NO_2_-Tyr into cytoskeletal proteins (Yemets *et al.*, 2011). Indeed, recently, *in vivo* evidence for extensive nitrotyrosination was provided by immunolocalization of N-Tyr along mitotic MT arrays in BY-2 cells (Blume *et al.*, 2013), indicating that N-Tyr might have specific affinity for highly dynamic MTs. Furthermore, a proteome approach identified the *A. thaliana* tubulin A6 as a target of nitrogen starvation, which leads to MT depolymerization in transgenic lines overexpressing GFP–TUA6 (Wang *et al.*, 2012). Finally, several tubulin isoforms were identified as putative *in vivo* targets of nitrotyrosination by immunopurification of proteins from *A. thaliana* seedlings with anti-nitrotyrosine antibodies and subsequent mass spectrometry (Lozano-Juste *et al.*, 2011).

Recent reports implicate that reactive oxygen species (ROS) signalling targets the MT cytoskeleton causing MT depolymerization, abnormal MT organization, and inhibition of cell cycle progression (Livanos *et al.*, 2012; Yao *et al.*, 2012). Also, depolymerization of MTs was observed following treatment with *Verticillium dahlia* toxins, which was shown to affect H_2_O_2_ and downstream NO homeostasis (Yao *et al.*, 2012). Finally, drug-induced disturbance of ROS homeostasis resulted in the formation of MT paracrystals and abnormally bent MT bundles (Livanos *et al.*, 2012). These reports support the direct modifications of the MT cytoskeleton by nitrosative stresses and the possible incorporation of N-Tyr into tubulin and MTs. However, in contrast to the above-mentioned studies, in the present study subtle and significant changes in the organization of the interphase MT array that were reversible were observed. Thus, evidence is provided that developmentally relevant low concentration NO signalling might act via delicately controlled modulation of MT organization. Consistently, exposure to SNP as an exogenous NO source also caused reorganization of the cortical MT array into randomly distributed MT bundles in *A. thaliana* epidermis cells of the differentiation zone (Yemets *et al.*, 2009), further correlating NO signalling and cytoskeletal organization. Intriguingly, guard cell function correlated with quantifiable changes in MT clustering or bundling within guard cells (Eisinger *et al.*, 2012a), and abscisic acid-induced guard cell aperture closure was accompanied by the reduction in MT structures (Eisinger *et al.*, 2012b). Although the causal relationship between NO signalling, which acts downstream of abscicic acid, and MT bundling in guard cell function has not been demonstrated, the experimental evidence is suggestive that this relationship indeed exists.

Structural modelling of the interaction between plant α-tubulin and dinitroanilines suggested that slight changes in tubulin primary structure could provoke changes in binding activity of dinitroanilines (Blume *et al.*, 2003). Combined treatment with oryzalin and NO_2_-Tyr showed multiplicative growth inhibition of BY-2 cells (Jovanovic *et al.*, 2010) and *Arabidopsis* (this study), supporting the notion that these drugs share a common target. Oryzalin binding to a conserved pocket of α-tubulin containing Thr239, just below the N-loop, might interfere with lateral binding to the M-loop of the adjacent dimer, thereby disrupting MTs and their polymerization (Morrissette *et al.*, 2004). Mutation of Thr239 to isoleucine in α-tubulin conferred resistance to oryzalin in goosegras (Mudge *et al.*, 1984), and expression of the respective tubulin mutant Thr239Ile in maize suspension culture also conferred oryzalin resistance (Antony *et al*., 1998). Oryzalin-induced MT depolymerization is diminished in the presence of NO_2_-Tyr, implying that NO_2_-Tyr might interfere with efficient oryzalin binding at its target site. Mutation of Tyr224 to alanine (Y224A) in *A. thaliana* TUA6, one of the tubulins that precipitated with anti-NO_2_-Tyr antibody (Lozano-Juste *et al.*, 2011), interferes with efficient incorporation into MTs when expressed in protoplasts, revealing the essential role of this tyrosine. Nevertheless, the observed effects of the Y224A mutant may not be related to failure of N-Tyr incorporation, but rather reflect other structural characteristics of TUA6. A similar observation in transgenic *A. thaliana* expressing the phosphomimic mutant TUA6^T349D^ (Fujita *et al.*, 2013) further demonstrates how post-translational tubulin modifications contribute to the establishment of the MT cytoskeleton.

Likewise, NO_2_-Tyr appeared to over-ride taxol effects on growth inhibition and MT organization. Taxol binding stabilizes the β-tubulin M-loop which interacts with N-loops from adjacent tubulin dimers to form lateral contacts (Snyder *et al.*, 2001; Morrisette *et al.*, 2004). Since potential nitrotyrosination could interfere with the conformation of the N-loop, lateral binding of dimers might be destabilized even in the presence of taxol, consistent with the present observations. As expected, expression of the GFP–TUA6 Y450A, replacing the C-terminal tyrosine, did not alter MT incorporation efficiency noticeably, consistent with the detection of detyrosinated tubulin isoforms along MTs in plants (Smertenko *et al.*, 1997).

Computer simulations that modelled the α-tubulin C-terminus in three potential configurations predicted the reorganization of the cortical MT cytoskeleton and changes in cell morphology as a consequence of conformational changes of the C-terminus (Blume *et al.*, 2005, 2013). According to these models, a C-terminal tyrosine would increase dynamicity of the MT polymer. In contrast, removal of the C-terminal tyrosine would cause conformational changes that could contribute to MT polymer stability. The addition of NO_2_-Tyr at the C-terminus would result in an intermediate MT polymer behaviour in this model. Consistent with the computer predictions, changes in MT organization and corresponding alterations of cell morphology were not observed upon NO_2_-Tyr treatment. Notably, simulation and modelling of the dynamics of tubulin C-termini also suggested that dynamic interactions between tubulin tails and the MT surface might influence MT polymerization and MT conformation, and thus flexibly alter the interaction of the MT with ligands (Freedman *et al.*, 2011). Indeed, removal of the α-tubulin C-terminal tail altered the efficiency and pH dependency of colchicine binding to tubulin (Chakraborty *et al.*, 2004). Similarly, the anions of glutamate-rich extreme C-termini of tubulin were involved in the regulation of vinblastine-induced tubulin polymerization (Rai and Wolff, 1998). Thus, NO_2_-Tyr at the C-terminus might lead to conformational changes of the tubulin dimer, and in consequence oryzalin and taxol binding might be inefficient, consistent with the present observations. Taken together, these findings further support the idea that NO signalling targets the MT cytoskeleton via post-translational modification of α-tubulin isoforms by direct incorporation of NO_2_-Tyr.

In animal systems it is well established that the balance between tyrosinated and detyrosinated α-tubulin in the MT cytoskeleton might reflect the developmental status of cells depending on whether they divide or differentiate (Quinones *et al.*, 2011). Within distinct MT subpopulations, entire filaments may consist of tubulin subunits containing α-tubulins predominantly in their tyrosinated or detyrosinated form. These subpopulations might also be associated with multiple other PTMs at the same time, adding to the level of regulation (Verhey and Gaertig, 2007; Quinones *et al.*, 2011). It has been recognized that stable MTs were associated with an increased degree of detyrosination, while highly dynamic MTs are mostly tyrosinated. Since MTs in most cells are usually very dynamic, the tyrosination cycle provides an effective way to modulate MT organization, for example during transitions between cell cycle phases (Janke and Kneussel, 2010).

In the experiments reported here, cortical MT array organization in the elongation zone appeared less well ordered upon low level NO_2_-Tyr treatment. MT arrays displayed fewer bundles and oblique angles relative to the longitudinal axis of the cell (Fig. 3) consistent with a recent study reporting the reorganization of cortical and endoplasmic MTs in the root apex, and the transition and elongation zone at concentrations >50 μM NO_2_-Tyr within 2h of treatment (Blume *et al.*, 2013). Although the concentration and treatment period vary, the present results are comparable with those of Blume *et al.* (2013).

The changes in cortical array organization could not be attributed to changes in MT plus-end dynamicity. However, analysis of a small data set suggested that frequencies of transitions between MT growth, shortening, and/or pausing might be altered upon NO_2_-Tyr treatment (Supplementary Fig. S4D at *JXB* online). Nevertheless, the present results are consistent with observations from guard cells, where the organization of MTs changed, despite unaltered MT dynamicity of the MT plus end (Eisinger *et al.*, 2012b). Changes in transition rates were also reported for a mutant of the MT plus-end binding protein AUGMIN subunit8 which is defective in MT reorganization, in addition to an altered MT plus-end shrinkage rate (Cao *et al.*, 2013), implying that changes in transition rates are regulated by MT-associated proteins (MAPs) and contribute to MT reorganization. It is proposed that MAPs might be responsible for the cortical MT array reorganization. Consistent with this idea, changes in cytosolic GFP–MBD were observed upon NO_2_-Tyr treatment (Supplementary Fig. S4E). Indeed, it was proposed that MAPs might be direct targets of PTMs, that act as road maps to regulate MAP and motor protein trafficking along MTs (Verhey and Gaertig, 2007).

In BY-2 cells, NO_2_-Tyr treatment readily altered the orientation of the division plane, and it was hypothesized that kinesins required for phragmoplast expansion and vesicle transport were specific targets of detyrosinated α-tubulins (Jovanović *et al.*, 2010). However, defects in phragmoplast expansion and vesicle transport typically result in incomplete cell walls and multinucleated cells (Jurgens, 2005) which were not observed in BY-2 cells (Jovanović *et al.*, 2010), indicating that mechanisms different from phragmoplast expansion and vesicle transport are the targets of NO signalling via incorporation of N-Tyr. Also, division plane orientations were altered in *A. thaliana* seedling root meristems upon long-term, low concentration NO_2_-Tyr treatment (Fig. 5). Nevertheless, NO_2_-Tyr treatment did not produce more pronounced defects in *pok* single and double mutants and did not induce hypersensitive responses in *pok* mutants, suggesting that the mechanism of phragmoplast guidance, which is disturbed in *pok* mutants (Müller *et al.*, 2006), was not specifically affected upon NO_2_-Tyr treatment. Thus, it is proposed that NO_2_-Tyr affects the MT cytoskeleton and associated MAPs already during prophase when the division plane is selected and established (Rasmussen *et al.*, 2011; Müller, 2012). Therefore, NO_2_-Tyr treatment might become a useful tool to induce oblique division planes to study the underlying molecular mechanism.

In summary, the results presented here support the hypothesis that incorporation of N-Tyr into the C-terminus of α-tubulins interferes with the α-tubulin tyrosination/detyrosination cycle, leading to MT reorganization via MAPs that differentially recognize PTMs at the C-terminus of α-tubulins. Thus, it is assumed that MAPs, which might be the prime targets of developmentally relevant NO signalling, transduce the NO signal by reorganizing the MT cytoskeleton.

## Supplementary data

Supplementary data are available at *JXB* online

Figure S1. (A) Reduction of root tip size. (B) Representative, inverted confocal images depict single optical sections of the root meristem.

Figure S2. Growth response curves of seedling roots under different control conditions.

Figure S3. Growth response of *A. thaliana* Columbia wild-type (Col wt) roots upon 3-nitro-l-tyrosine (NO_2_-Tyr) treatment.

Figure S4. Quantification of organization and dynamic of cortical microtubule (MT) arrays in epidermal cells at the elongation zone.

Figure S5. (A) Alignment of TUA6 isoforms. (B) 3D model of the nucleotide-binding site in α-tubulin.

Figure S6. Growth responses to 3-nitro-l-tyrosine (NO_2_-Tyr) in *A. thaliana* seedlings grown on standard medium for 4 d and subsequent exposure to different NO_2_-Tyr treatments for an additional 6 d.

Supplementary Data
