# Supplementary material for: Nitrosative stress triggers microtubule reorganization in Arabidopsis thaliana
Source: J Exp Bot. 2014 May 6;65(15):4177–89. doi: 10.1093/jxb/eru194 (PMC4112629; doi:10.1093/jxb/eru194)
Supplement: Supplementary Data [file supp_65_15_4177__index.html]

Nitrosative stress triggers microtubule reorganization in Arabidopsis thaliana — Nitrosative stress triggers microtubule reorganization in Arabidopsis thaliana — Supplementary Data 

# Nitrosative stress triggers microtubule reorganization in *Arabidopsis thaliana*

## Supplementary Data

Data files

**Files in this Data Supplement:**

- Supplementary Data - Supplementary Data
