# Supplementary material for: Nitrosative stress triggers microtubule reorganization in Arabidopsis thaliana
Source: J Exp Bot. 2014 May 6;65(15):4177–89. doi: 10.1093/jxb/eru194 (PMC4112629; doi:10.1093/jxb/eru194)
Supplement: Supplementary Data [file supp_eru194_jexbot121277_file001.pdf]

Nitrosative stress triggers microtubule reorganization in *Arabidopsis thaliana*

Elisabeth Lipka and Sabine Müller*

Supplemental Figure 1. (A) Reduction of root tip size. The distance between root tip and first root hair is significantly shorter in 3-nitro-L-tyrosine (NO_2-Tyr) treated plants (*P < 0.001, n=10) compared to control plants (n=10). **(B)** Representative, inverted confocal images depict single optical sections of the root meristem. On average multiple mitotic microtubule (MT) arrays are visible in single optical sections of control meristems. Considerably fewer mitotic MT arrays are present in single sections of NO_2-Tyr treated samples (see also Figure 1C). Mitotic figures (preprophase band, spindle, phragmoplast) in cross section are indicated by arrows. Scale bar is 20 µM.

Supplemental Figure 2. Growth response curves of seedling roots under different control conditions. Seedlings were grown on standard medium for four days and subsequently subjected to different treatments for 1.5 additional days. **(A)** Different 3-nitro-L-tyrosine (NO_2-Tyr) concentrations were compared with tyrosine (Tyr) concentrations supplemented in the medium. While seedlings grown on NO_2-Tyr showed dramatic decrease in growth rate, the corresponding Tyr controls showed a steady growth rate, similar to seedlings grown on medium supplemented with different HCl concentrations as mock (solvent only) control (n ≥ 27 plants). The lower abscissae represent different Tyr or NO_2-Tyr concentrations while the abscissae on the top, indicates HCl concentrations. **(B)** HCl supports the stability of the NO_2-Tyr solution, but is not a widely used solvent. To ensure that HCl by itself had no effect on plant growth we compared plant growth on plates prepared from NO_2-Tyr stock solution dissolved in 1 mM HCl or dissolved in DMSO. No significant differences were observed (n ≥ 19 plants) indicating that HCl had no effect.

Supplemental Figure 3. Growth response of *A. thaliana* Columbia wild type (Col wt) roots upon 3-nitro-L-tyrosine (NO_2-Tyr) treatment. **(A)** Comparison of root growth of Col wt (black, continuous line) and the microtubule reporter line GFP-MBD (red, dashed line). No significant differences were observed during the treatment and the overall trend of growth inhibition is similar for Col wt and the GFP-MBD line (n ≥ 19

plants for each data point). Seedlings were germinated and grown on standard medium for four days. Then root growth was evaluated (day 0) immediately before treatment with different concentrations of NO_2-Tyr (0 µM, 0.1 µM, 0.25 µM, 0.5 µM and 1 µM) started. After 1.5 days (day 1.5) and six days (day 6) of treatment root growth was measured and blotted over time. Note that root length between Col wt and GFP-MBD differ before treatment (day 0), but that slopes of growth rate are very similar, indicating that growth response to NO_2-Tyr treatment is comparable between Col wt and GFP-MBD.**(B)** Root tip morphology of Col wt plants on different NO_2-Tyr concentrations after six days of treatment.

Supplemental Figure 4. (A) Representative fluorescence intensity(arbitrary units, a.u.) profile plots, along the yellow lines in (A) are depicted as green line with squares for 0.5 µM HCl (control) and continuous black line for 0.5 µM NO_2-Tyr treated samples (the y-axis is shifted by 50 arbitrary units). Intensity values are background subtracted. **(B)** The distribution of intensity peaks into six fluorescence intensity classes for intensity blot profile in (B) is depicted. NO_2-Tyr treatment reduced the frequency of fluorescence peaks (black bars) throughout the different intensity classes (x-axis) compared to the control (green boxed bar). Values for NO_2-Tyr treatment are 7 (0-40), 15 (41-80), 12 (81-120), 5 (121-160), 1 (161-200) and 0 (>201). Values for controls are 11 (0-40), 45 (41-80), 42 (81-120), 33 (121-160), 14 (161-200) and 4 (>201). **(C)** Mean values for total numbers of MT bundles (defined as peaks) per µm in control (n = 9 cells from 6 roots) and treated samples (**P*<0.05, n = 10 cells from 6 roots). Error bars indicate ± standard deviation. **(D)** Growth (polymerization) and shortening (de-polymerization) velocities for microtubule (MT) plus ends and transition rates. No significant differences in MT dynamicity were observed between controls (n= 13 microtubules) and 0.5 µM NO_2-Tyr treated (n= 23 microtubules) cells. Transition frequencies between growth, shortening and pausing increase to 0.96 transitions/min in 0.5 µM NO_2-Tyr compared to 0.57 transitions/min in controls. Error bars indicate ± standard deviation. **(E)** Increase of cytosolic GFP-MBD determined by increase of fluorescent signal intensity upon increasing NO_2-Tyr concentrations. Number of cells and roots are indicated

(*P≤0.001).

Supplemental Figure 5. (A) Alignment of TUA6 isoforms. *Arabidopsis thaliana* (*A. th.*) TUA6 (At4g14960), goosegrass TUA1 (AAC05717), *Zea mays* TUA1 (P14641), Toxoplasma gondii TUA1 (AAA30145). Identical amino acids are indicated by dots. Green boxes indicate peptide sequences identifying *At.h.* TUA6 as a target of nitro-tyrosination (Lozano-Justo *et al.*, 2011). Blue boxes indicate helix 7 (H7) and M-loop which build the oryzalin binding pocket (Morrisette *et al.*, 2004). Mutation of Threonin 239 to Isoleucin in the H7 confers oryzalin resistance in goosegrass (Mudge *et al.,* 1984) and *Zea mays* (Anthony *et al.* 1998). Mutation in tyrosine 224 (Y224A) and tyrosine 450 (Y450A) mutants used in this study are indicated. Alignment was created with Alignment tool in CLC Main Workbenc 6.8.4. **(B)** 3D model (CDD structure model, http://www.ncbi.nlm.nih.gov/Structure/cdd/cddsrv.cgi?uid=100015), of nucleotide binding site in alpha tubulin. Positions of Y224 and amino acids which confer oryzalin resistance when mutated are labelled in yellow. Note that Y224 maps to the same face of H7 as mutated amino acids identified in oryzalin resistant lines.

Supplemental Figure 6. Growth responses to 3-nitro-L-tyrosine (NO_2-Tyr) are similar for wild type, *pok1* and *pok2* single and *pok1pok2* double mutants. As in previously described experiments, the *A. thaliana* seedlings were grown on standard medium for four days but then exposed to different NO_2-Tyr treatments for 6 additional days. **(A)** The decline in root growth upon different concentrations of NO_2-Tyr treatment does not differ significantly for GFP-MBD (indicated by blue triangle and continuous line) plants and two different allele combinations of *pok1pok2* double mutants (*pok1-1pok1-2* indicated by red circle and dotted line and *pok1-2pok2-2* indicated by green square and dashed line). The number of plants analyzed for each data point is n ≥ 15. **(B)** *pok* sinlge mutants and GFP-MBD control showed similar growth response to 0.5 µM NO_2-Tyr treatment. GFP-MBD is indicated by blue triangle and continuous line, *pok1-2* is indicated by the red circle and dotted line

and *pok2-1*;GFP-MBD (*pok2-1* in GFP-MBD background) is indicated by green square and dashed line. The number of plants analyzed for each genotype is n ≥ 15. Root length on control plates is set to 100% for each genotype.

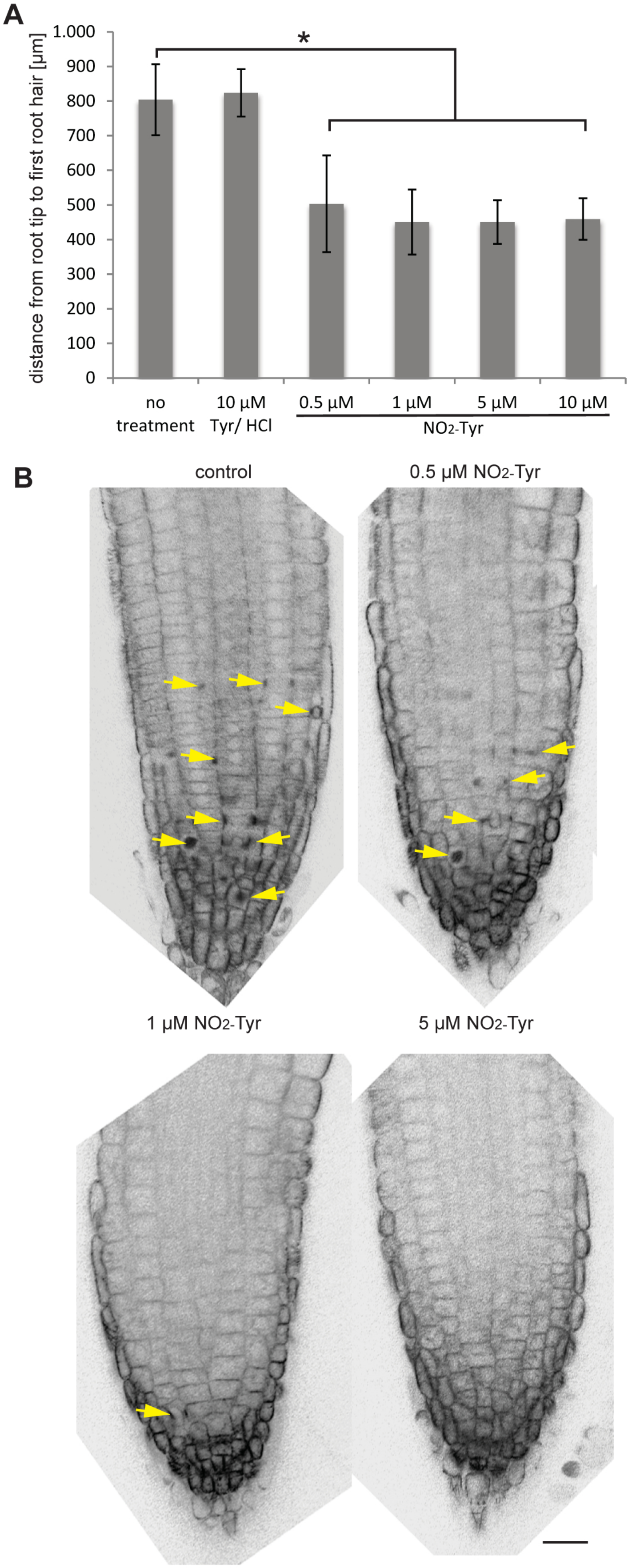

Supplementary Figure 1

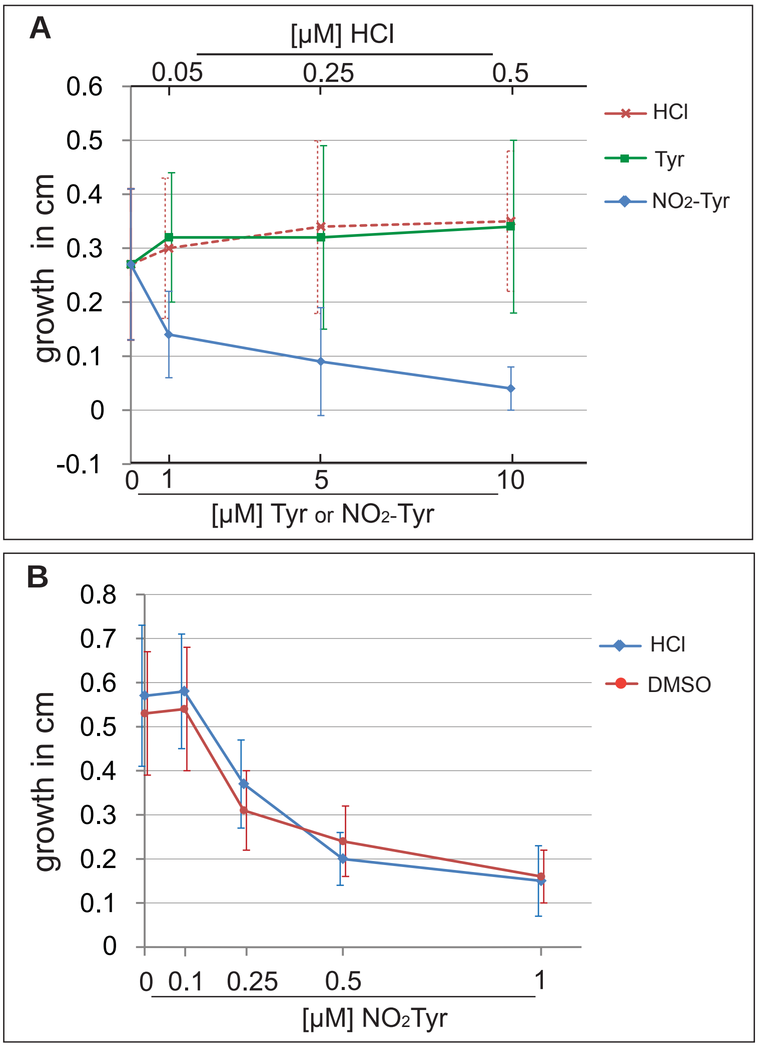

Supplementary Figure 2.

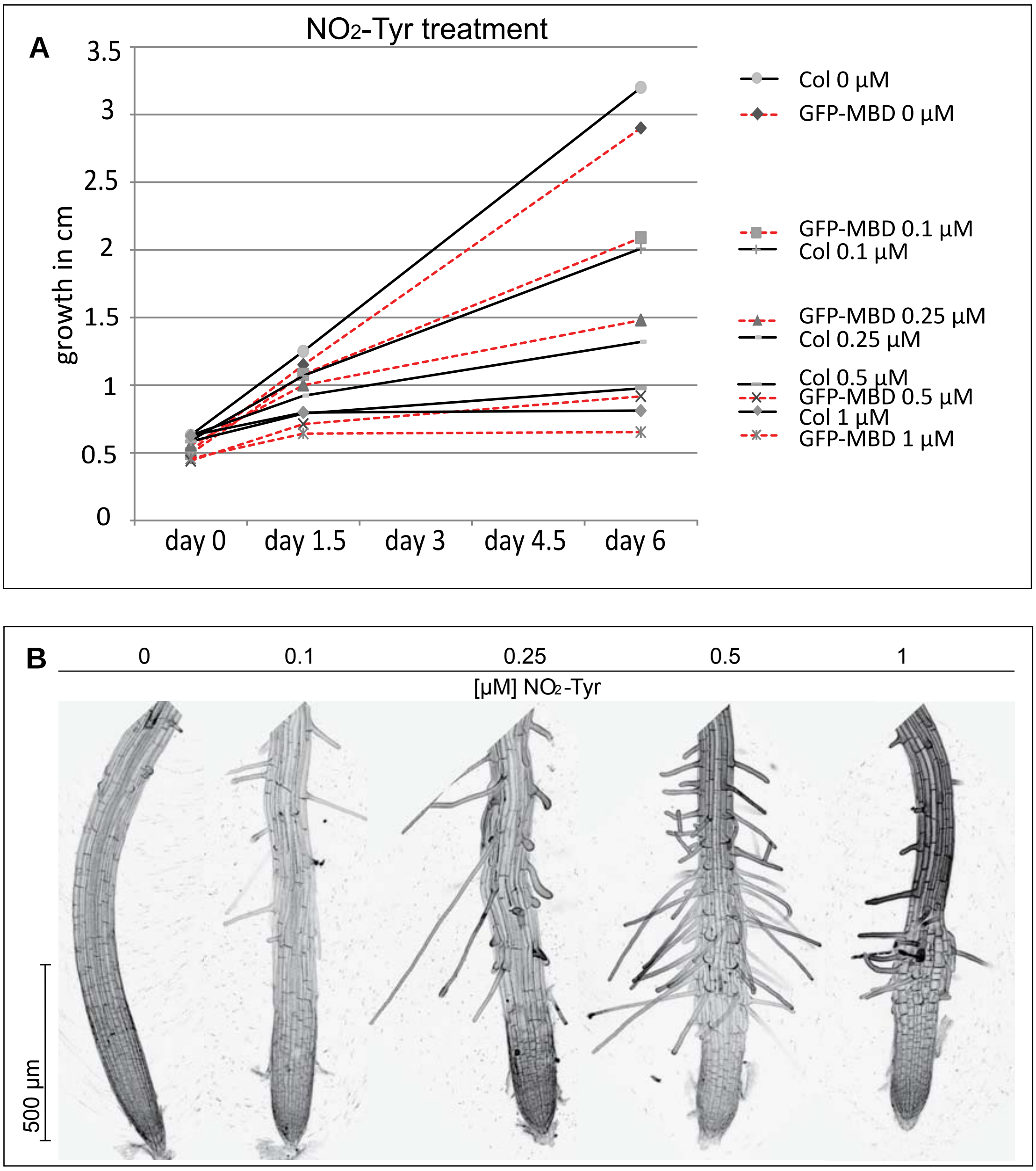

Supplementary Figure 3.

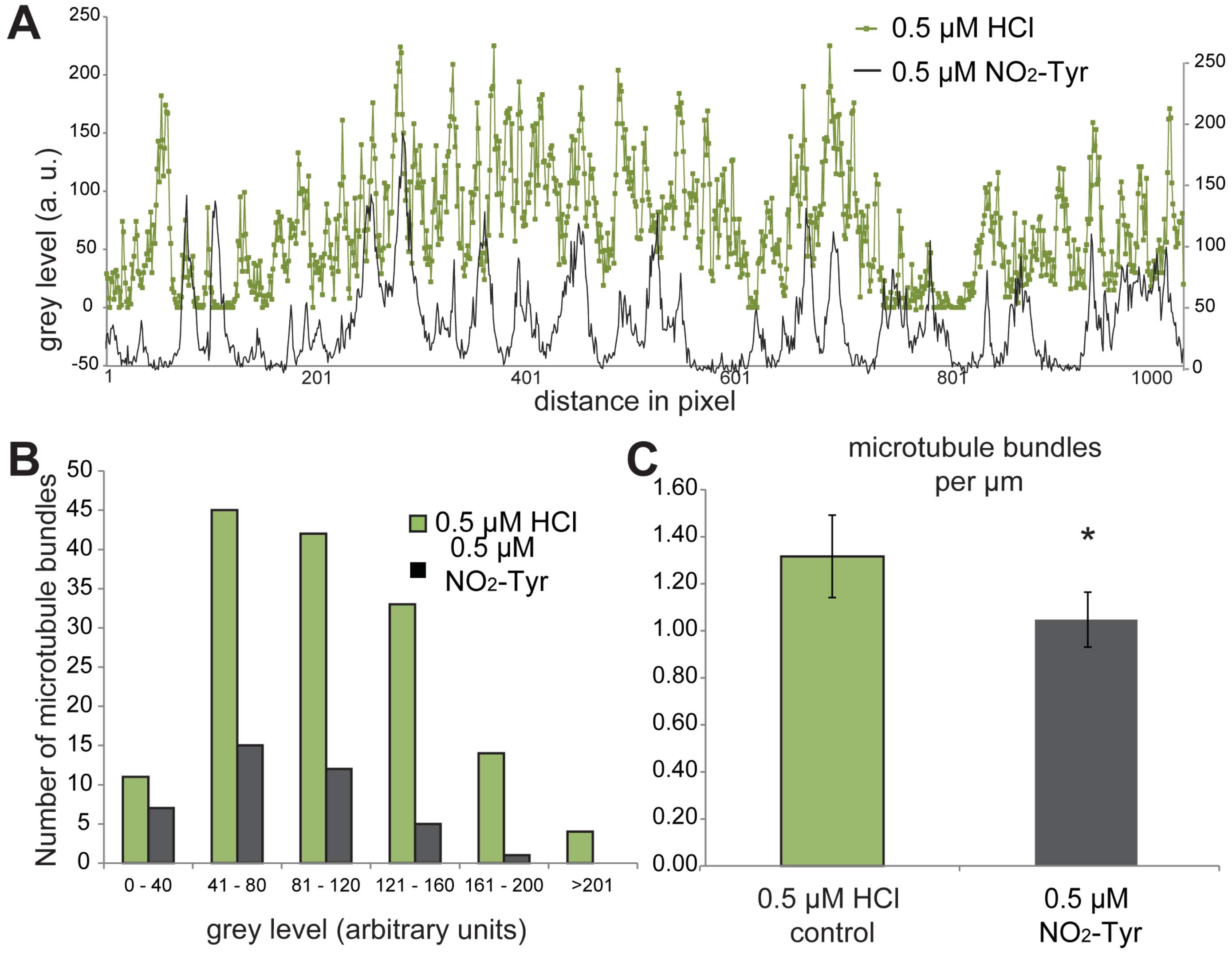

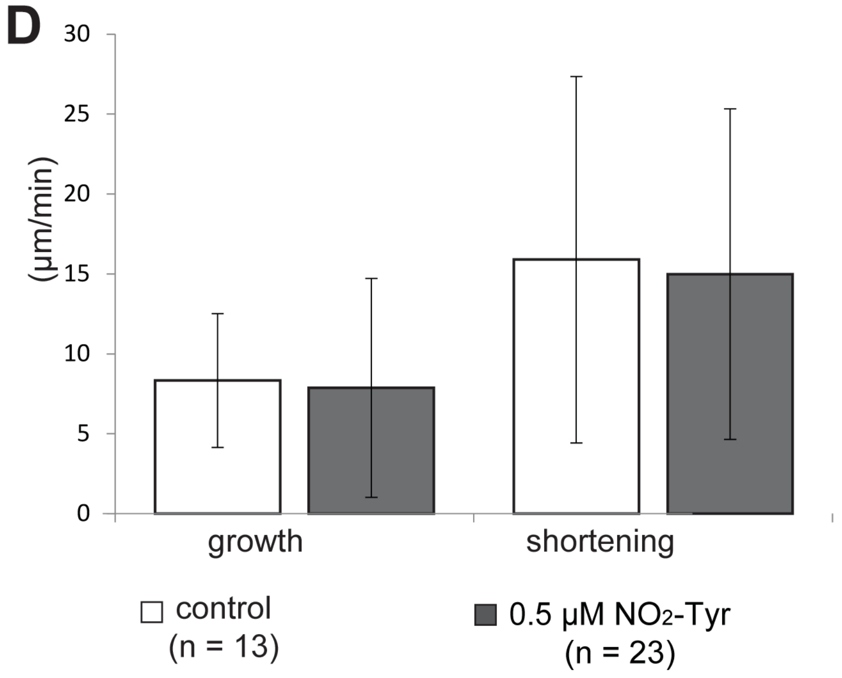

	control	0.5 µm NO2-Tyr
transition /min	0,57	0,96

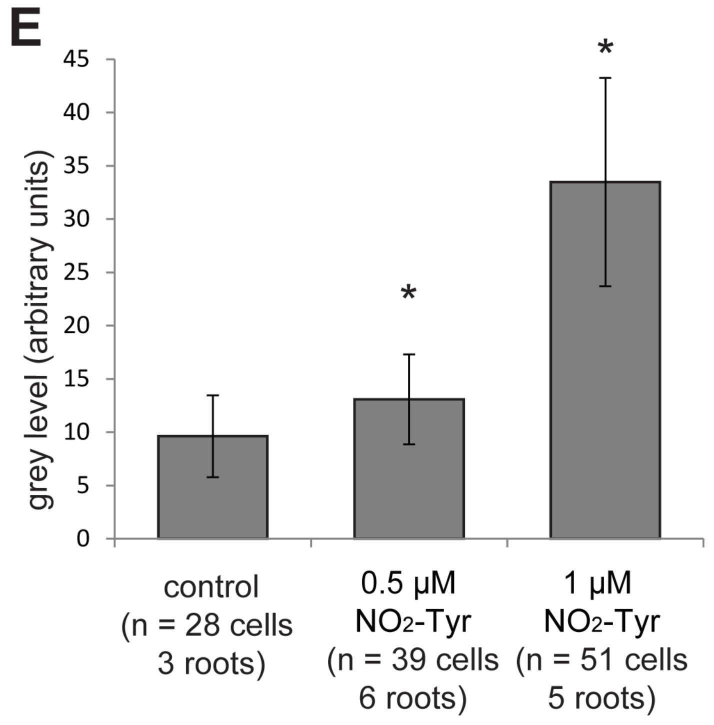

Supplementary Figure 4

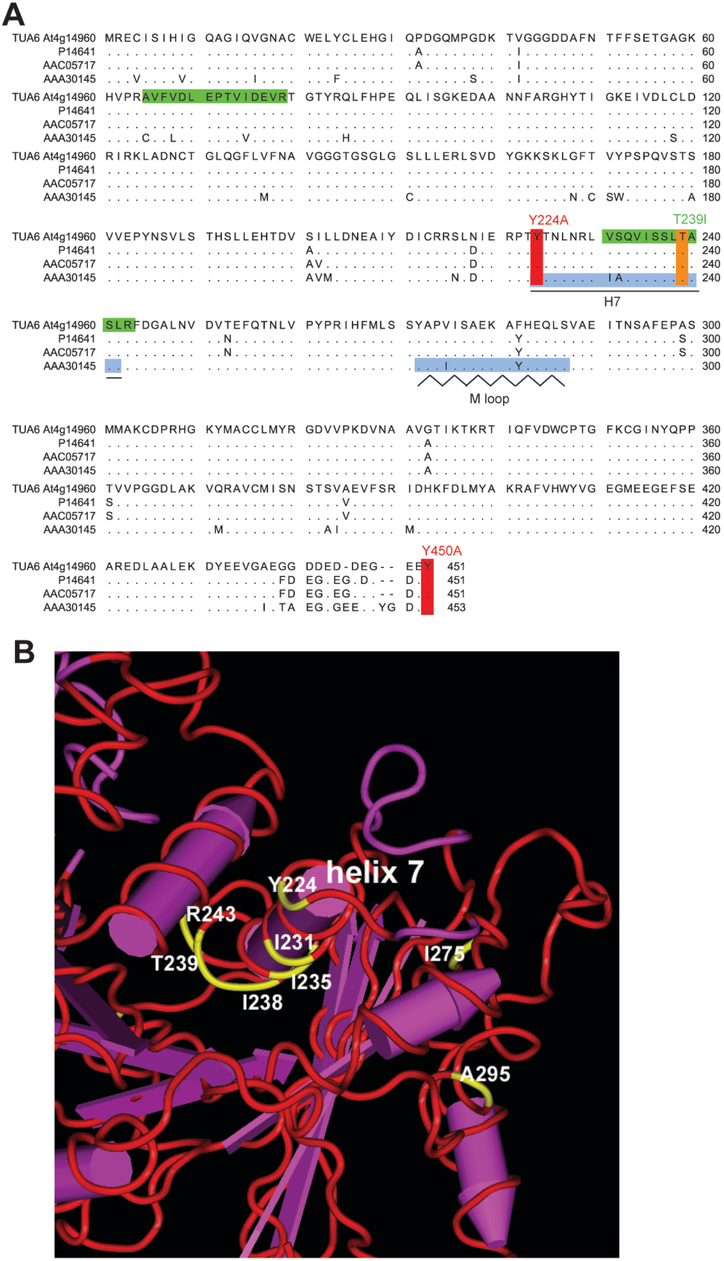

Supplementary Figure 5

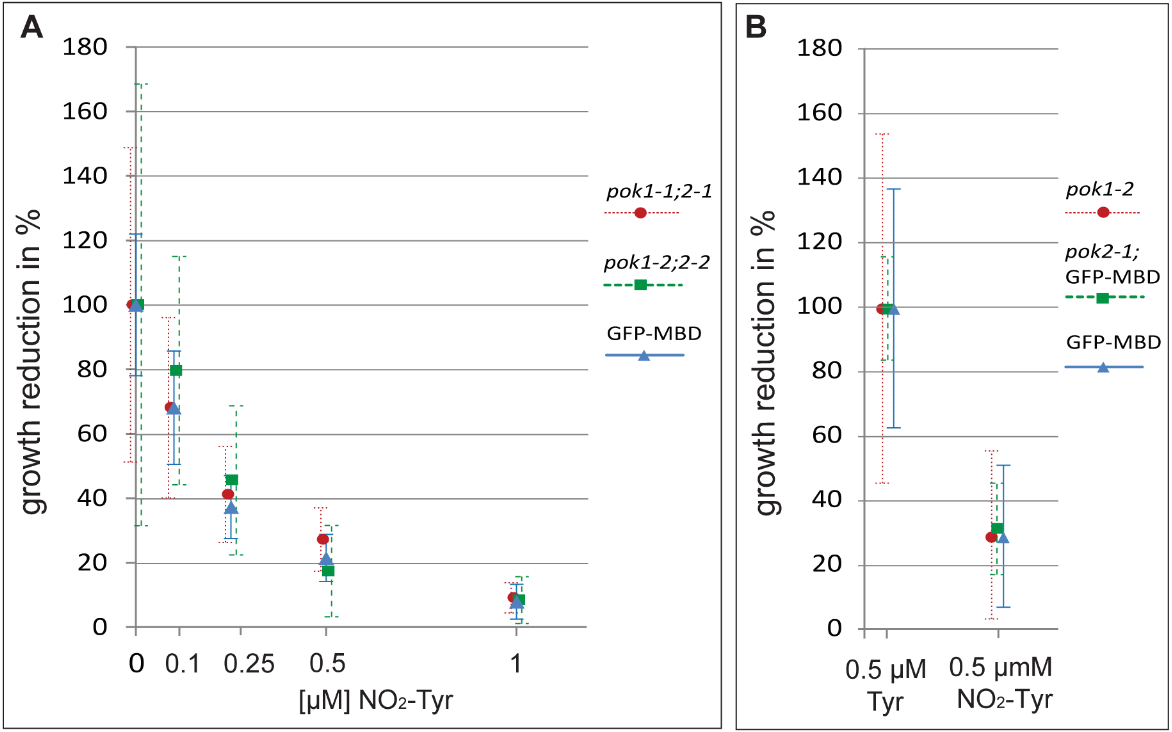

Supplementary Figure 6
